# Pituitary stalk interruption syndrome is characterized by genetic heterogeneity

**DOI:** 10.1371/journal.pone.0242358

**Published:** 2020-12-03

**Authors:** Raja Brauner, Joelle Bignon-Topalovic, Anu Bashamboo, Ken McElreavey

**Affiliations:** 1 Fondation Ophtalmologique Adolphe de Rothschild and Université Paris Descartes, Paris, France; 2 Human Developmental Genetics Unit, Institute Pasteur, Paris, France; Centre Hospitalier Universitaire Sainte-Justine, CANADA

## Abstract

Pituitary stalk interruption syndrome is a rare disorder characterized by an absent or ectopic posterior pituitary, interrupted pituitary stalk and anterior pituitary hypoplasia, as well as in some cases, a range of heterogeneous somatic anomalies. A genetic cause is identified in only around 5% of all cases. Here, we define the genetic variants associated with PSIS followed by the same pediatric endocrinologist. Exome sequencing was performed in 52 (33 boys and 19 girls), including 2 familial cases single center pediatric cases, among them associated 36 (69.2%) had associated symptoms or syndromes. We identified rare and novel variants in genes (37 families with 39 individuals) known to be involved in one or more of the following—midline development and/or pituitary development or function (*BMP4*, *CDON*, *GLI2*, *GLI3*, *HESX1*, *KIAA0556*, *LHX9*, *NKX2-1*, *PROP1*, *PTCH1*, *SHH*, *TBX19*, *TGIF1*), syndromic and non-syndromic forms of hypogonadotropic hypogonadism (*CCDC141*, *CHD7*, *FANCA*, *FANCC*, *FANCD2*, *FANCE*, *FANCG*, *IL17RD*, *KISS1R*, *NSMF*, *PMM2*, *SEMA3E*, *WDR11*), syndromic forms of short stature (*FGFR3*, *NBAS*, *PRMT7*, *RAF1*, *SLX4*, *SMARCA2*, *SOX11*), cerebellum atrophy with optic anomalies (*DNMT1*, *NBAS*), axonal migration (*ROBO1*, *SLIT2*), and agenesis of the corpus callosum (*ARID1B*, *CC2D2A*, *CEP120*, *CSPP1*, *DHCR7*, *INPP5E*, *VPS13B*, *ZNF423*). Pituitary stalk interruption syndrome is characterized by a complex genetic heterogeneity, that reflects a complex phenotypic heterogeneity. Seizures, intellectual disability, micropenis or cryptorchidism, seen at presentation are usually considered as secondary to the pituitary deficiencies. However, this study shows that they are due to specific gene mutations. PSIS should therefore be considered as part of the phenotypic spectrum of other known genetic syndromes rather than as specific clinical entity.

## Introduction

Pituitary stalk interruption syndrome (PSIS) is a rare disorder characterized by the combination of specific findings in magnetic resonance imaging (MRI) including an absent or ectopic posterior pituitary, absent or interrupted pituitary stalk and anterior pituitary hypoplasia [1]. This triad can be incomplete. The consequences of PSIS are a series of anterior pituitary deficiencies including growth hormone (GH) deficiency that may be isolated or may be associated with other hormonal deficiencies including thyroid-stimulating hormone, adrenocorticotropic and/or luteinizing hormone/follicle stimulating hormone. Posterior pituitary deficiency leading to central diabetes insipidus is very rare in PSIS. The clinical presentation of PSIS is heterogeneous and can include hypoglycemia, seizures, jaundice, micropenis, cryptorchidism or later in age decreased growth rate [2]. PSIS can be associated with other congenital anomalies, mainly ophthalmic, which can be isolated or as a part of syndromes, as well as intellectual disability or epilepsy [2–6]. The latter are considered as secondary to hypoglycemic episode due to GH and adrenocorticotropic deficiencies but they are frequently (more frequent and more marked) than that due to the GH and adrenocoticotropic deficiencies.

Familial forms represent around 5% of the cases [7]. It is mainly in these forms that mutations have been identified in genes mainly associated with pituitary development including *LHX4*, *OTX2*, *HEX1*, *SOX3*, *PROKR2*, *GPR161* [4,8–15]. Recently we identified mutations in the *CDON* and *ROBO1* genes in patients with PSIS and ophthalmic anomalies [16,17].

Here, we used an exome sequencing approach to identify the underlying genetic causes of PSIS in 52 including 2 familial patients monitored for PSIS by the same pediatric endocrinologist. We identified pathogenic and potentially pathogenic mutations in a wide range of genes known to be involved in a wide range of biological processes.

### Patients, family members, and controls

Written, informed consent was given by all the parents and by the patients aged more than 18 years for the clinical-biological evaluation (included in their hospital medical record) and for the molecular biology analyses with the use of protocols approved by local and national research ethics committees (Comité de Protection des Personnes Ile de France III, n°3445). The pituitary evaluation and follow-up were conducted as previously described [2].

This retrospective single-center study was performed in 52 individuals (33 boys and 19 girls). This includes 2 families each with 2 affected children. PSIS patients monitored for hypothalamic-pituitary deficiency by a senior pediatric endocrinologist (R. Brauner) in a university hospital between 1978 and 2018 and for whom a DNA sample was available. The 7 patients in whom a mutation has been previously found (patients 7, 8, 10, 17 and 37, Table 1) and published (*ROBO1*, *CDON*, *HEX1*) were included in this study [11,16,17].

**Table 1:** Phenotypes of 37 families of PSIS carrying potentially pathogenic genetic variants.

Case	Ancestry	Sex	Age at diagnosis (y)	Initial symptom(s)	Pituitary anomalies	Associated phenotypes	Genitalia/Puberty	MRI features
1	Afro-American	M	4	Hypoglycemia, jaundice	T, C, PRL, HH	Epilepsy, severe intellectual deficiency	Micropenis	Left temporal dysplasia, no differentiation between white and black cerebral matter
2	Indo-European	M	1.3	Hypoglycemia	T, C, partial HH	Ptosis	Cryptorchidism	Chiari
3	European	M	6.4	Decreased GR	None	None		
4	African	M	8.3	DI	T,DI, prepubertal	Optic nerve atrophy		No sella turcica nor pituitary, splenium of corpus callosum hypoplasia, ethmoïd meningocele, septum lucidum cyst
5	European	M	3.1	Decreased GR	T, prepubertal	Fanconi syndrome with microphtalmia	Cryptorchidism	
6	European	F	Neonate	Hypoglycemia, jaundice	T, C, HH	Cystic fibrosis		Corpus callosum atrophy, arachnoid cyst
7	European	M	1.0	Decreased GR	None	Left ptosis		
8*	European	F/F	3.9	Decreased GR	T, partial HH	Strabismus, transient cardiomyopathy	Secondary amenorrhea	
9	European	F	4.2	Hypoglycemia, seizures	T, C, HH	Optic nerve hypoplasia, epilepsy, intellectual deficiency		
10**	European	M/F	2.6	Decreased GR	None	Hypermetropia with divergent strabismus		
11	European	M	2.7	Hypoglycemia	T, C, HH	Cerebellar ataxia		Cerebellar hypoplasia
12	European	M	Neonate	Hypoglycemia	T, C, HH	None	Micropenis	
13	European	M	4.8	Decreased GR	None	Cleft lip and palate with hypoplasia hemi premaxillary bone		
14	European	M	5.8	Decreased GR	T, prepubertal	Intellectual deficiency	Cryptorchidism with very small testis	
15	European	M	6.7	Decreased GR	None	Bladder exstrophy, ano-rectal malformation	Cryptorchidism	
16	African	M	0.4	Jaundice	None, prepubertal	Fanconi syndrome, duodenal diaphragm, radial and thumb hypoplasia, microcornea, unic pelvic kidney, interventricular shunt	Cryptorchidism	Delay in myelinisation,
17	North African	F	Neonate	Hypoglycemia, jaundice, seizures	T, C, prepubertal	None		
						Mother: ptosis		
18	North African	F	3.5	Decreased GR	T, partial HH	Cystic teratoma on right ovary	Secondary amenorrhea	
19	East African	F	Neonate	Hypoglycemia	T	Strabismus		Agenesis interventricular septum and corpus callosum
20	European	M	Neonate	Hypoglycemia	T, C, HH	Strabismus, equinus foot deformity L	Micropenis, cryptorchidism	Abnormal signal in white matter of the brain
21	European	M	4.5	Decreased GR	None, prepubertal	Deafness	Cryptorchidism	
22	European	M	Neonate	Hypoglycemia, jaundice, seizures	T, C, HH	Major dysphagia	Cryptorchidism	
23	European	F	1.5	Hypoglycemia, decreased GR	T, C, HH	Intellectual delay, major obesity		
24	European	F	3.8	Intellectual delay, Hypoglycemia	T, C, HH	Severe intellectual deficiency and obesity, no language, seizures, choreoathetosis, thyroid dysfunction with basal and stimulated increased TSH		Absence of sella turcica
						Father: pulmonary dysfunction		
25	North African	M	2.8	Decreased GR	T, C, partial HH	Diabetes mellitus, peripheral hypothyroidism, Father died suddenly when young	Cryptorchidism, spermatogenic failure (inhibin B 0, micropenis and small testis)	
26	West African	M	Neonate	Hypoglycemia, jaundice	T, C, HH	Strabismus	Spermatogenic failure (inhibin B 0, micropenis, small testis)	
27	North African	F	9	Decreased GR	Partial HH	None	Secondary amenorrhea	Post pituitary as a nodule in the stalk
28	North African	M	5.6	Decreased GR	None	None		
						Mother: kidney failure by nephroangiosclerosis		
29	European	F	3	Decreased GR	None	Normal puberty but unexplained low inhibin B suggesting ovarian insufficiency		
30	European	M	5.5	Decreased GR	None	None		
31	European	M	3.5	Decreased GR	None, early puberty	Learning difficulties, pharyngeal abnormality, hypertrophic piloric stenosis	Cryptorchidism	
32	North African	M	3.4	Decreased GR	None	None		Temporal arachnoid cyst
33	European	M	2.6	Decreased GR	None, prepubertal	Learning difficulties		Chiari, syringomelia
34	African	F	2	Decreased GR	T, partial C, HH	Unilateral papillary hypoplasia, strabismus		
35	European	F	4.2	Decreased GR	None	None		
36	Africa	M	2.1	Decreased GR	T, C, HH	Single median incisor		
37	European	M	3.5	Decreased GR	None		Micropenis	

Abbreviations: Deficiency of T, thyrotropin, C adrenocorticotropic, G gonadotropin hormones; DI diabetes insipidus, PRL prolactin; HH hypogonadotropic hypogonadism (all without anosmia); GR: Growth rate.

*Affected sibs,

**Affected Aunt.

## Methods

Hypoglycemia was defined as a blood glucose concentration below 3 mmol/L after 2 days of age. Decreased growth rate was defined as a height velocity during the previous year of more than one standard deviation score below the mean for chronological age or decrease in height standard deviation of more than 0.5 over 1 year in children older than 2 years. Micropenis was defined as a penis length of less than 30 mm.

The criterion for diagnosing GH deficiency was a GH peak response of less than 20 mU/L or 6.7 ng/mL after two pharmacological stimulation tests or during spontaneous hypoglycemia, excluding the response to GH-releasing hormone, with low insulin-like growth factor 1 concentration. As we used various GH assays over the study period, we expressed the GH peak concentration in mU/L using conversion factors (ng/mL to mU/L) that were specific of the international standard used to calibrate the GH assay.

Thyroid-stimulating hormone deficiency was diagnosed by plasma free thyroxin below 12 pmol/L. Adrenocorticotropic deficiency was diagnosed by basal plasma cortisol concentrations at 8 a.m. below 40 ng/mL (110 nmol/L) in neonates and below 80 ng/mL (220 nmol/L) in older children, with no increase during hypoglycemia and low/normal adrenocorticotropic concentration. Gonadotropin deficiency was diagnosed by the absence of pubertal development at 13 years in girls and 14 years in boys and no or partial gonadotropins response to a gonadotropin-releasing hormone stimulation test [18]. The plasma osmolalities were measured after water deprivation for 12 hours in 30 patients with concomitant urinary osmolality in 23 of them. All were normal (275 to 300 mosmol/kg in the plasma and between 700 and 1300 mosmol/kg in the urinary) except in one case who presented with diabetes insipidus.

The follow-up for each patient included measurements of plasma free thyroxin and cortisol concentrations at 8 a.m. every one or two years, if their concentrations had previously been normal to diagnose delayed deficiency.

### Exome sequencing and array-CGH analysis

Exon enrichment was performed as described elsewhere using Agilent SureSelect Human All Exon V4 [19]. Paired-end sequencing was performed on the Illumina HiSeq2000 platform with an average sequencing coverage of x50. Read files were generated from the sequencing platform via the manufacturer’s proprietary software. Reads were mapped using the Burrows–Wheeler Aligner and local realignment of the mapped reads around potential insertion/deletion (indel) sites was carried out with the GATK version 1.6. SNP and indel variants were called using the GATK Unified Genotyper for each sample. SNP novelty was determined against dbSNP138. Datasets were filtered for novel or rare (MAF<0.01) variants. Novel and rare variants were analyzed by a range of web-based bioinformatics tools using the EnsEMBL SNP Effect Predictor (http://www.ensembl.org/homosapiens/userdata/uploadvariations). All variants were screened manually against the Human Gene Mutation Database Professional [Biobase] (http://www.biobase-international.com/product/hgmd). *In silico* analysis was performed to determine the potential pathogenicity of the variants using Polyphen (http://genetics.bwh.harvard.edu/pph), and SIFT (http://sift.jcvi.org/www/SIFT_chr_coords_submit.html) online tools that predict the effect of human mutations on protein function. We focused our analyses on non-synonymous coding, nonsense, and splice site variants, filtering out all known common variations contained in dbSNP (build 138) (www.ncbi.nlm.nih.gov/projects/SNP/), the 1000 Genomes Project (http://www.1000genomes.org/)and in the gnomAD database (http://gnomad.broadinstitute.org/). An in-house database of 700 exomes from control individuals or individuals with unrelated pathologies were also screened for the potential pathogenic variants identified in the PSIS cohort. Variants were confirmed by visual examination using the IGV browser or by Sanger sequencing. Variants were classified according to ACMG guidelines [20]. In the vast majority of cases the parent’s DNA was unavailable for study, therefore trio analysis was not possible. Exome datasets were also compared to an in-house control dataset of >700 exomes. Karyotyping was performed using standard methods and chromosomes were observed after G and R banding. FISH analysis was carried out using FITC or rhodamin labeled probes localized in the chromosomal breakpoints regions. For the array-CGH, genome wide copy number analysis was performed using Illumina CytoSNP12 BeadChip arrays (Illumina, San Diego, California, USA). The samples were processed using the Infinium assay and results analyzed by Illumina Genome Studio software.

## Results and discussion

The age at diagnosis of PSIS index cases ranged from birth to 10.8 years (Table 1). Among the 52 patients, there was consanguinity in two cases (cases 27 and 28) and the father deceased suddenly at 45 years in case 25. The initial symptom leading to the diagnosis of PSIS was hypoglycemia in 18 (34.6%) cases, seizures with concomitant hypoglycemia in 3 (5.7%), jaundice in 6 (11.5%), and/or decreased growth rate in 30 (57.7%). By MRI all patients had an ectopic posterior pituitary gland, except 6 patients where it was not seen and one with small posterior pituitary associated with interrupted stalk (case 1). Thus, the pituitary stalk was defined as interrupted (n = 19 with a nodule in case 27), not observed (n = 21), thin (n = 8), normal (n = 3) and large (n = 1). The sagittal median anterior pituitary height was <1 mm or not seen in 9 patients. The associated symptoms or syndromes (36 cases, 69.2%) are detailed in the Table 1. Ophthalmic malformations are present in 16 cases (30.8%).

The GH deficiency was isolated in 21 cases (40.4%), or associated with isolated thyrotropin deficiency in 4 cases (7.7%) or multiple deficiencies including gonadotropins deficiency in 23 (44.2% or 56.1% after excluding 11 in prepubertal age). Only one patient had diabetes insipidus (case 4). Two patients had early puberty. Three girls (cases 8, 18 and 27) had secondary amenorrhea, associated with thyrotropin deficiency, after normal pubertal development, despite a normal pubertal gonadotropins response to gonadotropin-releasing hormone test. These were considered as having partial gonadotropins deficiency [18].

The heterogeneity of the clinical presentation of patients with PSIS is explained by the wide variety of the genes carrying potentially pathogenic variants. In 39 individuals we identified genetic variants, which may contribute to the complex phenotypes seen in this series of patients (Table 2). Array-CGH analysis indicated normal ploidy and did not indicate changes in gene copy number associated with the phenotypes. However, exome sequencing identified rare and novel variants in genes known to be involved in one or more of the following—midline development and/or pituitary development or function (*BMP4*, *CDON*, *GLI2*, *GLI3*, *HESX1*, *KIAA0556*, *LHX9*, *NKX2-1*, *PROP1*, *PTCH1*, *SHH*, *TBX19*, *TGIF1*), syndromic and non-syndromic forms of hypogonadotropic hypogonadism (HH; *CCDC141*, *CHD7*, *FANCA*, *FANCC*, *FANCD2*, *FANCE*, *FANCG*, *IL17RD*, *KISS1R*, *NSMF*, *PMM2*, *SEMA3E*, *WDR11*), syndromic forms of short stature (*FGFR3*, *NBAS*, *PRMT7*, *RAF1*, *SLX4*, *SMARCA2*, *SOX11*), cerebellum atrophy with optic anomalies (*DNMT1*), axonal migration (*ROBO1*, *SLIT2*), and agenesis of the corpus callosum (*ARID1B*, *CC2D2A*, *CEP120*, *CSPP1*, *DHCR7*, *INPP5E*, *VPS13B*, *ZNF423*). In the majority of cases, these variants were also absent from in-house controls or present at a very low frequency (Table 2).

**Table 2:** Gene variants in 37 families associated with PSIS.

Case	Gene	Variant	ZY	mutation	Predicted effect on protein	dbSNP	ACMG classification	MAF and population (GnomAD)	In-house controls (MAF)	MI	Associated phenotypes (OMIM)	PV	GV	PR
1	*PROP1*	NM_006261:c.63delG:p.L21fs	het	frameshift	loss-of-function	rs780134343	Pathogenic	0.0000961; African	Absent	AR	Pituitary hormone deficiency, combined, 2 (262600)		**44**	
	*IL17RD*	NM_017563:c.1256T>C:p.I419T	het	missense	SIFT, deleterious; PolyPhen2, probably damaging; REVEL 0.644	rs145388838	Likely pathogenic	0.0001; NFE	Absent	AR/AD	Hypogonadotropic hypogonadism 18 with or without anosmia (606807)			
	*SMARCA2*	NM_001289396:c.787T>A:p.S263T	het	missense	SIFT, deleterious; PolyPhen2, probably damaging; REVEL 0.029	Novel	Likely pathogenic	NA	Absent	AD	Nicolaides-Baraitser syndrome (601358)			
2	*GLI3*	NM_000168.5:c.4180C>T:p.R1394C	het	missense	SIFT, deleterious; PolyPhen2, benign; REVEL 0.091	rs577664817	Uncertain significance	0.00036; South Asian	Absent	AD	Greig cephalopolysyndactyly syndrome (175700); Pallister-Hall syndrome (146510); Polydactyly, postaxial, types A1 and B (174200); Polydactyly, preaxial, type IV (174700); Hypothalamic hamartomas, somatic (241800)		**43**	
	*IL17RD*	NM_017563:c.794C>G:p.P265L	het	missense	SIFT, deleterious; PolyPhen2, possibly damaging; REVEL. 0.432	rs759628358	Likely pathogenic	0.000158; South Asian	Absent	AR/AD	Hypogonadotropic hypogonadism 18 with or without anosmia (606807)			
3	*SHH*	NM_000193.3:c.52G>T:p.V18L	het	missense	SIFT, tolerated; PolyPhen2, unknown; REVEL 0.349	rs148181557	Uncertain significance	0.000096; African	Absent	AD	Holoprosencephaly 3 (142945); Microphthalmia with coloboma 5 (611638); Schizencephaly (269160); Single median maxillary central incisor (147250)		**45**	
	*CHD7*	NM_017780:c.6377A>T:p.D2126V	het	missense	SIFT, deleterious; PolyPhen2, probably damaging; REVEL 0.345	rs1064794182	Uncertain significance	NA	Absent	AD	CHARGE syndrome (214800); Hypogonadotropic hypogonadism 5 with or without anosmia (612370)		**43**	
4	*NBAS*	NM_015909:c.1083+4C>T	hom	essential splice site	Loss-of-function	rs112852390	Uncertain significance	0.014; African	0.0032	AR	Infantile liver failure syndrome 2 (616483); Short stature, optic nerve atrophy, and Pelger-Huet anomaly (614800)			
	*KIAA0556*	NM_015202:c.3346+8G>T	het	essential splice site	Loss-of-function	rs374277288	Uncertain significance	0.0007; African	0.001	AR	Joubert syndrome 26 (616784)		**46**	
	*KIAA0556*	NM_015202:c.2180A>T:p.H727L	het	missense	SIFT, deleterious; PolyPhen2, benign; REVEL 0.047	rs139943989	Uncertain significance	0.0011; African	Absent	AR	Joubert syndrome 26 (616784)		**46**	
	*ROBO1*	NM_002941:c.1565G>A:p.R522Q	het	missense	SIFT, tolerated; PolyPhen2, benign; REVEL 0.139	rs138082446	Likely pathogenic	0.0061; African	Absent	AD	NA		**17**	
5	*CHD7*	NM_017780:c.6476C>A:p.S2159Y	het	missense	SIFT, deleterious; PolyPhen2, probably damaging; REVEL 0.202	Novel	Uncertain significance	NA	Absent	AD	CHARGE syndrome (214800); Hypogonadotropic hypogonadism 5 with or without anosmia (612370)		**43**	
	*FANCA*	NM_001286167:c.3971C>T:p.P1324L	het	missense	SIFT, deleterious; PolyPhen2, probably damaging; REVEL 0.714;	rs182657062	Pathogenic	0.0001; NFE	Absent	AR	Fanconi anemia, complementation group A (227650)			
	*FANCA*	NM_001286167:c.1193_1196del:p.V398fs	het	frameshift	Loss-of-function	Novel	Pathogenic	NA	Absent	AR	Fanconi anemia, complementation group A (227650)			
	*GLI3*	NM_000168:c.1346GG>A:p.R449Q	het	missense	SIFT, tolerated; PolyPhen2, benign; REVEL 0.175	rs745809543	Uncertain significance	0.000045; NFE	Absent	AD	Greig cephalopolysyndactyly syndrome (175700); Pallister-Hall syndrome (146510); Polydactyly, postaxial, types A1 and B (174200); Polydactyly, preaxial, type IV (174700); Hypothalamic hamartomas, somatic (241800)		**43**	
	*SEMA3E*	NM_012431:c.C1498T:p.R500W	het	missense	SIFT, deleterious; PolyPhen2, probably damaging; REVEL 0.488	rs111300014	Uncertain Signficance	0.0001; EAS	Absent	AD	CHARGE syndrome (214800)			
	*SLX4 (FANCP)*	NM_032444:c.C3143T:p.S1048F	het	missense	SIFT, deleterious; PolyPhen2, probably damaging; REVEL 0.174	Novel	Uncertain Significance	NA	Absent	AR	Fanconi anemia, complementation group P (613951)			
6	*NBAS*	NM_015909:c.G6311A:p.R2104Q	het	missense	SIFT, tolerated; PolyPhen2, benign; REVEL 0.204	rs773412024	Uncertain Significance	0.000097; African	Absent	AR	Infantile liver failure syndrome 2 (616483); Short stature, optic nerve atrophy, and Pelger-Huet anomaly (614800)			
	*NBAS*	NM_015909:c.T1118C:p.L373P	het	missense	SIFT, deleterious; PolyPhen2, probably damaging; REVEL 0.211	Novel	Uncertain Significance	NA	Absent	AR	Infantile liver failure syndrome 2 (616483); Short stature, optic nerve atrophy, and Pelger-Huet anomaly (614800)			
	*CFTR*	NM_000492:exon11:c.1520_1522del:p.507_508del	hom	Deletion	NA	rs113993960	Pathogenic	0.0106; NFE	0.0034	AR	Cystic fibrosis (219700)			
7	*ROBO1*	NM_002941:c.G3450T:p.Y1150X	het	nonsense	Loss-of-function	Novel	Pathogenic	NA	Absent	AD	NA	**17**	**17**	
8*	*ROBO1*	NM_002941:c.G719C:p.C240S	het	missense	SIFT, deleterious; PolyPhen2, probably damaging; REVEL 0.542	Novel	Pathogenic	0.0001076; African	Absent	AD	NA	**17**	**17**	
9	*WDR11*	NM_018117:c.T109G:p.Y37D	het	missense	SIFT, deleterious; PolyPhen2, probably damaging; REVEL 0.347	rs776728184	Likely pathogenic	0.00003; NFE	Absent	AD	Hypogonadotropic hypogonadism 14 with or without anosmia (614858)		**13**	
	*PMM2*	NM_000303:c.254_255del:p.Q85fs	het	frameshift	Loss-of-function	Novel	Uncertain Significance	NA	Absent	AR	Congenital disorder of glycosylation, type Ia (212065)			
10**	*ROBO1*	NM_002941:c.2928_2929delG, p.A977Qfs	het	Frameshift	Loss-of-function	Novel	Pathogenic	NA	Absent	AD	NA	**17**	**17**	
11	*DNMT1*	NM_001130823.3:c.A2858G:p.D953G	het	Missense	SIFT, deleterious; PolyPhen2, probably damaging; REVEL 0.780	Novel	Likely pathogenic	NA	Absent	AD	Cerebellar ataxia, deafness, and narcolepsy, autosomal dominant (604121); Neuropathy, hereditary sensory, type IE (614116)			
12	*NSMF*	NM_001130969.1:c.C53A:p.S18X	het	Nonsense	Loss-of-function	Novel	Likely pathogenic	NA	Absent	AD	Hypogonadotropic hypogonadism 9 with or without anosmia (614838)			
13	*ARID1B*	NM_020732:c.A5015T:p.N1672I	het	Missense	SIFT, deleterious; PolyPhen2, probably damaging; REVEL 0.610	Novel	Uncertain significance	NA	Absent	AD	Coffin-Siris syndrome 1 (135900)		**47**	
	*VPS13B*	NM_017890:c.C4298G:p.S1433C	het	Missense	SIFT, deleterious; PolyPhen2, probably damaging; REVEL 0.532	Novel	Uncertain significance	NA	Absent	AR	Cohen syndrome (216550)			
14	*LHX9*	NM_020204:c.T2C:p.M1T	het	Missense	SIFT, deleterious; PolyPhen2, probably damaging; REVEL 0.603	rs201066309	Uncertain significance	0.00008637; AMR	Absent	NA	NA			
	*INPP5E*	NM_019892:c.G907A:p.V303M	het	Missense	SIFT, deleterious; PolyPhen2, possibly damaging; REVEL 0.699	rs746212325	Pathogenic	0.00003472; NFE	Absent	AR	Joubert syndrome 1 (213300); Mental retardation, truncal obesity, retinal dystrophy, and micropenis (610156)		**43**	**25**
15	*BMP4*	NM_130851:c.C1001T:p.A334V	het	missense	SIFT, deleterious; PolyPhen2, possibly damaging; REVEL 0.905	rs550409227	Uncertain significance	0.00001499; NFE	Absent	AD	Microphthalmia, syndromic 6 (607932); Orofacial cleft 11 (600625)		**43**	
	*SLX4 (FANCP)*	NM_032444:c.G248C:p.G83A	het	missense	SIFT, tolerated; PolyPhen2, benign; REVEL 0.048	rs771698977	Uncertain significance	0.00005994; NFE	Absent	AR	Fanconi anemia, complementation group P (613951)			
16	*CDON*	NM_001243597:c.A1343G:p.H448R	het	missense	SIFT, deleterious; PolyPhen2, probably damaging; REVEL 0.119	rs1209875838	Uncertain significance	NA	Absent	AD	Holoprosencephaly 11 (614226)		**16**	
17	*GLI2*	NM_005270:c.G2455A:p.V819M	het	missense	SIFT, deleterious; PolyPhen2, probably damaging; REVEL -	Novel	Uncertain significance	NA	Absent	AD	Culler-Jones syndrome (615849); Holoprosencephaly 9 (610829)		**43**	
	*PTCH1*	NM_000264:c.G3929A:p.G1310D	het	missense	SIFT, deleterious; PolyPhen2, probably damaging; REVEL 0.348	Novel	Uncertain significance	NA	Absent	AD	Holoprosencephaly 7 (610828)			
	*WDR11*	NM_018117:c.G3571A:p.G1191S	het	missense	SIFT, deleterious; PolyPhen2, probably damaging; REVEL 0.692	rs149486212	Likely pathogenic	0.0002; NFE	Absent	AD	Hypogonadotropic hypogonadism 14 with or without anosmia (614858)		**13**	
18	*CDON*	NM_001243597:c.T2764C:p.E922X	het	nonsense	Loss-of-function	Novel	Pathogenic	NA	Absent	AD	Holoprosencephaly 11 (614226)	**16**		
19	*CHD7*	NM_017780:c.G7085A:p.S2362N	het	missense	SIFT, deleterious; PolyPhen2, probably damaging; REVEL 0.120	rs139876661	Uncertain significance	0.0023; African	Absent	AD	CHARGE syndrome (214800); Hypogonadotropic hypogonadism 5 with or without anosmia (612370)		**43**	
	*GLI2*	NM_005270:c.G598A:p.A200T	het	missense	SIFT, tolerated; PolyPhen2, benign; REVEL 0.171	rs111840592	Uncertain significance	0.0045; African	Absent	AD	Culler-Jones syndrome (615849); Holoprosencephaly 9 (610829)		**43**	
20	*FANCG*	NM_032656:c.C748T:p.Q86X	het	nonsense	Loss-of-function	Novel	Likely pathogenic	NA	Absent	NA	Fanconi anemia, complementation group G (614082)			
	*SLX4 (FANCP)*	NM_032444.3:c.G4445A:p.C1482Y	het	missense	SIFT, deleterious; PolyPhen2, probably damaging; REVEL 0.024	rs148856258	Uncertain significance	0.00032; African	Absent	AR	Fanconi anemia, complementation group P (613951)			
21	*FANCD2*	NM_0033044:c.1277_1278+5del	het	essential splice site	Loss-of-function	Novel	Uncertain significance	NA	Absent	AR	Fanconi anemia, complementation group D2 (227646)			
	*RAF1*	NM_002880:c.A1756T:p.A586S	het	missense	SIFT, deleterious; PolyPhen2, probably damaging; REVEL 0.372	Novel	Uncertain significance	NA	Absent	AD	Cardiomyopathy, dilated, 1NN (615916); LEOPARD syndrome 2 (611554); Noonan syndrome 5 (611553)			
22	*CCDC141*	NM_173648:c.A2183G:p.N728S	het	missense	SIFT, tolerated; PolyPhen2, possibly damaging; REVEL 0.044	rs151185557	Uncertain significance	0.0001; NFE	Absent	AR	NA		**43**	
23	*TBX19*	NM_005149:c.603+6->GTGTTTGT	homo	essential splice site	Loss-of-function	Novel	Uncertain significance	NA	Absent	AR	Adrenocorticotropic hormone deficiency (201400)			
24	*PRMT7*	NM_019023:c.T1480C:p.W494R	het	missense	SIFT, deleterious; PolyPhen2, possibly damaging; REVEL 0.598	rs751670999	Pathogenic	0.000086; AMR	Absent	AR	Short stature, brachydactyly, intellectual developmental disability, and seizures (617157)			**36**
	*NKX2-1*	NM_001079668:c.G67C:p.G23R	het	missense	SIFT, tolerated; PolyPhen2, probably damaging; REVEL 0.332	rs773410433	Likely pathogenic	0.00003286; South Asian	Absent	AD	Choreoathetosis, hypothyroidism, and neonatal respiratory distress (610978)		**41**	
	*SOX11*	NM_003108:c.C885G:p.D295E	het	missense	SIFT, deleterious; PolyPhen2, probably damaging; REVEL NA	Novel	Uncertain significance	NA	Absent	AD	Mental retardation, autosomal dominant 27 (615866)			
25	*TGIF1*	NM_170695:c.T25C:p.S9P	het	missense	SIFT, deleterious; PolyPhen2, probably damaging; REVEL 0.078	rs148390122	Uncertain significance	0.0057; African	Absent	AD	Holoprosencephaly 4 (142946)		**45**	
	*FANCC*	NM_000136:c.G137A:p.R46K	het	missense	SIFT, tolerated; PolyPhen2, benign; REVEL 0.062	rs765058606	Uncertain significance	0.00001499; NFE	Absent	AR	Fanconi anemia, complementation group C (227645)			
26	*FGFR3*	NM_001163213:c.875A>T: p.E292V	het	missense	SIFT, deleterious; PolyPhen2, probably damaging; REVEL 0.494	Novel	Uncertain significance	NA	Absent	AD	Achondroplasia (100800); CATSHL syndrome (610474); Crouzon syndrome with acanthosis nigricans (612247); Hypochondroplasia (146000); LADD syndrome (149730); Muenke syndrome (602849); SADDAN (616482); Thanatophoric dysplasia, type I (187600); Thanatophoric dysplasia, type II (187601)			
27	*KIAA0556*	NM_015202:c.G4836C:p.E1612D	het	missense	SIFT, tolerated; PolyPhen2, possibly damaging; REVEL 0.086	rs775146768	Uncertain significance	0.00008639; AMR	Absent	AR	Joubert syndrome 26 (616784)		**46**	
	*CSPP1*	NM_024790:c.A1972G:p.R658G	het	missense	SIFT, deleterious; PolyPhen2, benign; REVEL 0.107	rs199996939	Uncertain significance	0.001; AMR	Absent	AR	Joubert syndrome 21 (615636)			**25**
28	*CHD7*	NM_017780:c.C1696G:p.P566A	het	missense	SIFT, deleterious; PolyPhen2, probably damaging; REVEL 0.088	rs764518030	Uncertain significance	0.0001172; Latino	Absent	AD	CHARGE syndrome (214800); Hypogonadotropic hypogonadism 5 with or without anosmia (612370)		**43**	
	*WT1*	NM_024426:c.C278G:p.A93G	het	missense	SIFT, deleterious; PolyPhen2, probably damaging; REVEL NA	Novel	Pathogenic	NA	Absent	AD	Denys-Drash syndrome (194080); Frasier syndrome (136680); Meacham syndrome (608978); Mesothelioma, somatic 156240);Nephrotic syndrome, type 4 (256370); Wilms tumor, type 1 (194070)			
	*WT1*	NM_024426:c.C250T:p.P84S	het	missense	SIFT, deleterious; PolyPhen2, probably damaging; REVEL 0.045	Novel	Pathogenic	NA	Absent	AD	Denys-Drash syndrome (194080); Frasier syndrome (136680); Meacham syndrome (608978); Mesothelioma, somatic 156240);Nephrotic syndrome, type 4 (256370); Wilms tumor, type 1 (194070)			
	*FANCE*	NM_021922:c.G1379A:p.R460Q	het	missense	SIFT, deleterious; PolyPhen2, probably damaging; REVEL 0.056	rs541746126	Uncertain significance	0.00009619; African	bsent	AR	Fanconi anemia, complementation group E (600901)			
29	*DHCR7*	NM_001163817:c.355delC:p.H119fs	het	frameshift	Loss-of-function	rs747827699	Pathogenicv	0.0000155; NFE	Absent	AR	Smith-Lemli-Opitz syndrome (270400)		**43**	
	*ZNF423*	NM_015069:c.T1144C:p.S382P	het	missense	SIFT, deleterious; PolyPhen2, probably damaging; REVEL 0.145	rs142835239	Uncertain significance	0.0014; NFE	Absent	AR/AD	Joubert syndrome 19 (614844); Nephronophthisis 14 (614844)			
	*FSHR*	NM_000145:c.G1451A:p.R484H	het	missense	SIFT, tolerated; PolyPhen2, benign; REVEL 0.250	rs763241241	Likely pathogenic	0.0001; EAS	Absent	AR/AD	Ovarian dysgenesis 1 (233300); Ovarian hyperstimulation syndrome (608115); Ovarian response to FSH stimulation (276400)			
30	*FGFR3*	NM_001163213:c.A2120G:p.K707R	het	missense	SIFT, deleterious; PolyPhen2, possibly damaging; REVEL 0.622	rs369813768	Uncertain significance	0.00001534; NFE	Absent	AD	Achondroplasia (100800); CATSHL syndrome (610474); Crouzon syndrome with acanthosis nigricans (612247); Hypochondroplasia (146000); LADD syndrome (149730); Muenke syndrome (602849); SADDAN (616482); Thanatophoric dysplasia, type I (187600); Thanatophoric dysplasia, type II (187601)			
	*CHD7*	NM_017780:c.A2185G:p.K729E	het	missense	SIFT, deleterious; PolyPhen2, probably damaging; REVEL 0.422	rs41272437	Uncertain significance	0.0013; AMR	Absent	AD	CHARGE syndrome (214800); Hypogonadotropic hypogonadism 5 with or without anosmia (612370)		**43**	
	*FANCD2*	NM_001018115:c.G3290A:p.R1097Q	het	missense	SIFT, deleterious; PolyPhen2, probably damaging; REVEL 0.372	rs755748094	Uncertain significance	0.00007493; NFE	Absent	AR	Fanconi anemia, complementation group D2 (227646)			
31	*CEP120*	NM_153223:c.A2114G:p.Y705C	het	missense	SIFT, tolerated; PolyPhen2, benign; REVEL 0.365	rs373838092	Uncertain significance	0.00006083; SAS	Absent	AR	Joubert syndrome 31 (617761); Short-rib thoracic dysplasia 13 with or without polydactyly (616300)			
32	*GLI2*	NM_005270.4:c.G2159A:p.R720H	het	missense	SIFT, deleterious; PolyPhen2, probably damaging; REVEL 0.446	rs149091975	Uncertain significance	0.001366; African	0.002	AD	Culler-Jones syndrome (615849); Holoprosencephaly 9 (610829)		**43**	
	*GLI2*	NM_005270.4:c.A538C:p.M180L	het	missense	SIFT, deleterious; PolyPhen2, benign; REVEL 0.249	rs565813552	Uncertain significance	0.0003388; Latino	Absent	AD	Culler-Jones syndrome (615849); Holoprosencephaly 9 (610829)		**43**	
	*SLIT2*	NM_004787.3:c.T3095C:p.L1032S	het	missense	SIFT, tolerated; PolyPhen2, probably damaging; REVEL 0.742	rs768269055	Uncertain significance	0.0001388; Other	Absent	NA	NA			
33	*CC2D2A*	NM_001080522:c.G2356A:p.E786K	het	missense	SIFT, deleterious; PolyPhen2, probably damaging; REVEL 0.266	Novel	Uncertain significance	NA	Absent	AR	COACH syndrome (216360); Joubert syndrome 9 (612285); Meckel syndrome (612284)		**43**	
	*WDR11*	NM_018117:c.199-9T>C	het	essential splice site	Loss-of-function	rs565141290	Uncertain significance	0.000045; NFE	Absent	AD	Hypogonadotropic hypogonadism 14 with or without anosmia (614858)		**13**	
34	*CCDC141*	NM_173648.3:c.C1402T:p.R468W	het	missense	SIFT, deleterious; PolyPhen2, possibly damaging; REVEL 0.075	rs550015011	Uncertain significance	0.007; African	0.0007	AD	NA		**43**	
	*KISS1R*	NM_032551.5:c.G710C:p.R237P	het	missense	SIFT, deleterious; PolyPhen2, probably damaging; REVEL	Novel	Likely pathogenic	NA	Absent	AR	Hypogonadotropic hypogonadism 8 with or without anosmia (614837)			
35	*KIAA0556*	NM_015202:c.G1232T:p.G411V	het	missense	SIFT, tolerated; PolyPhen2, benign; REVEL 0.080	rs201073350	Uncertain significance	0.0002; NFE	Absent	AR	Joubert syndrome 26 (616784)		**46**	
36	*GATA5*	NM_080473.4:c.C56G:p.S19W	het	missense	SIFT, deleterious; PolyPhen2, probably damaging; REVEL 0.732	rs200383755	Uncertain significance	0.005443; NFE	0.0014	AR/AD	Congenital heart defects, multiple types, 5 (617912)			
37	*HESX1*	NM_003865.2:c.G445A:p.E149K	het	missense	SIFT, deleterious; PolyPhen2, probably damaging; REVEL 0.937	rs104893742	Pathogenic	0.00005016; East Asian	Absent	AR/AD	Pituitary hormone deficiency, combined, 5 (182230)	**11**		**11**

**Abbreviations**. ZY zygosity, MI reported mode of inheritance, PV variant previously associated with the PSIS (reference), GV gene variants previously associated with the PSIS (reference), PR, variant previously reported in association with a mendelian disorder (reference), NFE non-finnish European, AMR mixed American, SAS South Asian, AR autosomal recessive, AD autosomal dominant, het heterozygous, hom homozygous, N/A not available.

Other variants, which contributed to the clinical phenotype of the patient, but not involved in PSIS include patient 6 with cystic fibrosis who is homozygous for the common deletion variant (del:p.507_508del), *WT1* variants (p.P84S, p.A93G) associated with a familial history of renal anomalies in family 28 and an FSHR variant (p.R484H) in patient 29 associated with low inhibin B levels suggestive of ovarian insufficiency.

Several patients presented with complex syndromes, where PSIS has not been previously reported. In several of these cases the phenotype may be due to a combination of gene variants rather than single variant. Patient 1 presented with severe early epilepsy and intellectual deficiency, pituitary deficiencies as well as a micropenis. MRI showed PSIS leading to the clinical diagnosis of PSIS and Nicolaides-Baraitser syndrome. This child carried pathogenic and likely pathogenic variants in *PROP1* and *IL17RD*, as well as a likely pathogenic variant in *SMARCA2*. Variants in latter are a known cause of Nicolaides-Baraitser syndrome [21] It is unclear if PSIS represents an extension of the phenotypic spectrum associated with the Nicolaides-Baraitser syndrome due to a *SMARCA2* variant or if the PSIS is due to the *PROP1* and/or *IL17RD* variants. Pituitary stalk anomalies have been reported in association with a homozygous, loss-of-function variant in *PROP1*, however the child described here is heterozygous for a LOF variant [22]. To resolve this, other patients with Nicolaides-Baraitser syndrome or hypogonadotropic hypogonadism will need to be screened for PSIS by MRI.

Patients 4 and 6 have biallelic, or homozygous variants in *NBAS*. Variants in *NBAS* are associated with autosomal recessive forms of either infantile liver failure syndrome 2 or short stature, optic nerve atrophy, and the Pelger-Huet anomaly [23,24]. Patient 4 presented with an undiagnosed syndromic form of short stature. Some aspects of the phenotype may be associated with the *NBAS* pathogenic variants including with diabetes insipidus at birth, left anophtalmia and optic nerve and chiasma agenesis. However, other features have not been reported in association with *NBAS* variants including the absence of the sella turcica and pituitary, hypoplasia of the splenium of the corpus callosum, ethmoïdal meningocele and a septum lucidum cyst. The child also carried two rare variants in the *KIAA0556* gene, where previously homozygous variants in *KIAA0556* have been reported in association with micropenis, pituitary hypoplasia, pituitary stalk anomalies, cleft palate and cerebellar hypoplasia in Joubert syndrome 26 [25]. To add further complexity to the interpretation of the data, the child also carries a rare missense variant in *ROBO1*. *ROBO1* variants are associated with PSIS and optic nerve anomalies [17,26,27]. It is possible that the complex phenotype may be due to contributions of each of these variants. However, the observation that patient 6 presented with neonatal hypoglycemia and jaundice due to acute liver failure and carried two rare *NBAS* variants implies that PSIS may be a part of the phenotypic spectrum associated with NBAS pathogenic variants [24]. The child also presented with cystic fibrosis due to a homozygous *CFTR* variant.

Patient 9 presented with a syndromic form of short stature consisting of hypoglycemia, seizures, hypothyroidism, hypogonadism and failure to thrive. MRI showed PSIS. Based on the clinical phenotype the child had an initial clinical diagnosis of GH and adrenocorticotropic deficiencies responsible for hypoglycemia and seizures and secondary intellectual deficiency. The girl carries a novel heterozygous frameshift variant in *PMM2*. However, congenital disorder of glycosylation, type 1a, which is caused by biallic variants in PMM2 is an autosomal recessive disorder and *PMM2* LOF variants are common in the general population ([28]; https://gnomad.broadinstitute.org/gene/ENSG00000140650?dataset=gnomad_r2_1). Thus, although aspects of the phenotype are consistent with congenital disorder of glycosylation the pathogenicity of the PMM2 p.Q85fs variant is uncertain. The girl also carries a predicted likely pathogenic rare missense variant in *WDR11*. WDR11 variants are associated with an autosomal dominant form of HH [29]. Recently a heterozygous p.I436V WDR11 variant was reported in a child with combined pituitary hormone deficiencies, a small anterior pituitary, ectopic posterior pituitary, and a thin, interrupted stalk [13]. This child also carried a loss-of-function PROKR2 (p.R85C) and the pituitary anomalies were considered to be due to both of these variants.

Patient 11 presented with a complex phenotype consisting of short stature, hypoglycemia and cerebellar ataxia with cerebellar hypoplasia. The patient was initially clinically diagnosed with two independent presentations: neurological features secondary to prematurity and perinatal anoxia and hypoglycemia and failure to thrive due hypothalamic pituitary deficiency. Exome sequencing revealed that the affected boy carries a novel missense variant, p.D953G, in the bromo-adjacent homology 2 (BAH2) domain of the methyltransferase DNMT1 [30]. The BAH2 domain is required for controlling the interaction of the methyltransferase with the DNA major groove [31]. Heterozygous DNMT1 pathogenic variants are associated with autosomal dominant neurodegenerative disorders affecting both the central and peripheral nervous systems. Interestingly, variants in exon 20 of DNMT1 leads to hereditary sensory and autonomic neuropathy type IE [32], and variants in exon 21 cause autosomal dominant cerebellar ataxia, deafness and narcolepsy. Both of these exons encode the replication focus targeting sequence (RFTS) domain [33]. Here, the boy presented with cerebellar ataxia with cerebellar hypoplasia, which was detected by MRI at 2.7 years. There is no evidence of either deafness or narcolepsy. The affected aspartic acid 953 residue is highly conserved in vertebrates. It is interesting to speculate that this phenotype, including PSIS, may be specifically due to pathogenic variants in this domain of the DNMT1 protein. Indeed, the DNMT1 protein has been shown to physically interact with HESX1, a protein which is essential for pituitary development, and it is co-expressed with Hesx1 during murine pituitary development [34]. Variants in HESX1 are associated with pituitary anomalies and we have previously described a variant in HESX1 associated with PSIS (case 37; 11).

Patient 21 presented with deafness diagnosed during the first year, cryptorchidism and then decreased growth rate. The clinical diagnosis was initially considered to be GH deficiency due to PSIS, which was considered to be independent of the deafness. However, the cryptorchidism was unexplained, as this was not due to gonadotropin deficiency. Analysis of the exome sequencing dataset revealed that he carried a novel RAF1 variant p.A586S located within the highly conserved region CR3. Pathogenic variants, including those located within the CR3 domain, are associated with a wide spectrum of phenotypes including Noonan syndrome 5, LEOPARD syndrome and non-syndromic cardiomyopathy [35]. Pathogenic RAF1 variants tend cluster in two regional 2 hotspots (CR2 ser259 or CR3 ser612). Although pathogenic variants in CR3 are usually not associated with cardiomyopathy, the contribution of the RAF1 p.A586S variant to the phenotype patient 21 is unclear.

Patient 24 presented with severe intellectual deficiency, hypoglycemia, obesity, seizures, thyroid dysfunction and choreoathetosis. The PSIS was diagnosed on the MRI performed in the evaluation of the intellectual deficiency. Despite the complete GH deficiency, this girl had spontaneous normal statural growth with adult height above the mean. This unusual feature has been reported to the reported hypoglycaemia leading to overconsommation of glucides and obesity. This girl carries a known pathogenic variant in PRMT7 [36]. Biallelic variants in PRMT7 are associated with an autosomal recessive form of short stature, brachydactyly, intellectual developmental disability, and seizures [36] However, this variant is heterozygous and it is unlikely to be responsible for the phenotype. However, the girl also carries rare or novel missense variants in *NKX2-1* and *SOX11*. SOX11 variants are associated with an autosomal dominant form of short stature with intellectual deficiency as part of the Coffin-Siris syndrome 9 [37,38]. However, all of the SOX11 pathogenic variants reported to date fall within the functional HM-box domain (amino acids 47–122). Variants outside the SOX11 HMG-box are associated with congenital anomalies of the kidney and urinary tract (CAKUT; [39]). Hence, we consider that the SOX11 variant is unlikely to be responsible for the phenotype. Pathogenic variants in NKX2-1 are associated with choreoathetosis, hypothyroidism, and neonatal respiratory distress syndrome [40]. The association of ppituitary anomalies with NKX2-1 variants has been reported rarely in the literature. However, both point mutations involving NKX2-1 as well as a deletion of the entire gene [41,42] have been reported with pituitary and/or pituitary stalk anomalies. An affected father and his daughter were reported with respectively low LH levels, leading to hypogonadism, or low GH levels, causing short stature. Both patients had motor developmental delay and chorea. Thyroid-stimulating hormone levels were normal in the affected daughter. Both cases carried nonsense variant in NKX2-1. Based on the similarity between this family and patient 24 we suggest that there is now evidence to support the inclusion of pituitary anomalies with NKX2-1.

Genetic variation in several of the genes reported here have been recently suggested to contribute to PSIS including *ARID1B*, *BMP4*, *CC2D2A*, *CCDC141*, *CDON*, *CHD7*, *DHCR7*, *GLI2*, *GLI3*, *INPP5E*, *KIAA0556*, *PROP1*, *PROKR2*, *SHH*, *TGIF1* and *WDR11* [13,16,17,43–48]. However, we identified new candidate genes for PSIS including seven families, who carried variants in genes known to be involved in Fanconi anemia (patients, 5, 15, 20, 21, 25, 28 and 30), although only one case (patient 5) presented with Fanconi syndrome and microphtalmia. These findings may not be surprising considering that a proportion of Fanconi anemia patients present with hormone deficiencies (GH deficiency, hypogonadism) and short stature [3,49]. Other novel findings include a rare SLIT2 missense variant with two rare GLI2 variants (patient 32). SLITs are a conserved family of secreted proteins that were originally discovered in the nervous system where they signal through ROBO receptors to mediate axonal guidance and branching [50,51]. SLIT2 is the ligand for ROBO1 that we and others have previously shown to be involved in PSIS suggesting a contribution to the development of the phenotype.

The most common genetic finding in this group was rare/novel variants associated with anomalies pituitary development and/or HH with variants observed in 14 of the 29 families (excluding the patients in prepubertal age). Of these 29 cases, 14 carried either heterozygous mutations in more than one gene or potentially biallelic mutations in the same gene. This is consistent with previous findings of autosomal dominant causes of pituitary anomalies and di- or oligogenic causes of HH [52]. In the entire cohort 17 of the patients were diagnosed with hypogonadotropic hypogonadism. Of these cases 6 did not harbor variants in genes known to cause HH. 11 of the 23 carried rare or novel variants in genes known to cause HH. Surprisingly, a further 6 patients with potentially pathogenic variants in HH genes did not present with HH.

These variants may explain the majority of the symptoms/syndromes presented by the patients included in this series and in other reported series. It is important to point out that it not possible to exclude that only one variant in patients carrying several variants is responsible for the full phenotype and that the remainder may have a minimal contribution to the phenotype. However, a proportion of patients are not explained by genetic variants in these genes. These cases may be due to mutations in other genes involved in pituitary development or function, which are currently unrecognized or may be due to variants in non-coding sequences including copy number variants that would not have been detected in this study. In some cases, described above, the clinical presentation is not fully explained by our current knowledge of biological function of the genes. However, our data expand the phenotypic spectrum associated with many of the genes described in this study and also suggest that PSIS may be associated with heterozygous carriers of autosomal recessive disorders. Similar findings were reported a large cohort of individuals with unexplained short stature [53]. Hauer et al., observed a heterozygous variant in FGFR3 in an individual, who at the initial clinical presentation had no obvious skeletal anomalies that are associated with pathogenic FGFR3 variants. Similarly, they found heterozygous variants in genes that were previously reported to cause autosomal recessive skeletal dysplasias [53]. In this study, we also observed heterozygous variants in genes reported to cause autosomal recessive disorders, which were associated with PSIS and atypical clinical presentations (e.g. case 24, *PRMT7*; case 29, *DHCR7*). As suggested by the authors, current descriptions of genotype-phenotype relationships are incomplete and the phenotypic spectrum needs to be expanded [53].

The initial presentation, leading to the diagnosis of PSIS, like seizures, intellectual disability, micropenis or cryptorchidism, are usually considered as secondary to the pituitary deficiencies. This study shows that they are likely due to pathogenic variants responsible for epilepsy, cerebral or cerebella developments, thyroid development or hypogonadotropic hypogonadism. In conclusion, the phenotypic heterogeneity seen in association with PSIS is reflected in a complex genetic heterogeneity. In many circumstances PSIS may be considered as part of the phenotypic spectrum of other known genetic syndromes rather than as specific clinical entity.
